# Driving Sustainability: Carbon Footprint, 3D Printing, and Legislation concerning Electric and Autonomous Vehicles

**DOI:** 10.3390/s23229104

**Published:** 2023-11-10

**Authors:** Mihailo Jovanović, Tomás de J. Mateo Sanguino, Milanko Damjanović, Milena Đukanović, Nikolas Thomopoulos

**Affiliations:** 1 Faculty of Management Herceg Novi, University Adriatik, Zemunska 143, 85348 Meljine, Montenegro; mihajovanovic30@gmail.com; 2 Escuela Técnica Superior de Ingeniería, Universidad de Huelva, Av. de las Artes, s/n, 21007 Huelva, Spain; 3 Faculty of Mechanical Engineering, University of Montenegro, Dzordza Vasingtona bb, 81000 Podgorica, Montenegro; milanko@ucg.ac.me; 4 Faculty of Electrical Engineering, University of Montenegro, Dzordza Vasingtona bb, 81000 Podgorica, Montenegro; milenadj@ucg.ac.me; 5 Department of Tourism and Transport, School of Hospitality and Tourism Management, University of Surrey, Guildford GU2 7XH, UK; n.thomopoulos@surrey.ac.uk

**Keywords:** electric and autonomous vehicles, environmental protection, carbon emissions, additive manufacturing, regulations

## Abstract

In recent years, there has been a remarkable development in the technology and legislation related to electric and autonomous vehicles (i.e., EVs/AVs). This technological advancement requires the deployment of the most up-to-date supporting infrastructure to achieve safe operation. Further infrastructure is needed for Level 5 vehicles, namely the introduction of super-fast wireless 5G technology. To achieve harmony between the rapid technological advancement of EVs/AVs and environmental preservation, enacting legislation related to their sustainable use is vital. Thus, this manuscript provides a review of the technological development of EVs/AVs, with a special focus on carbon footprints and the implementation of additive manufacturing using recycled materials. While EVs have a 12.13% increased carbon footprint compared to conventional vehicles, AVs with basic and advanced intelligence features have an increased carbon footprint of 41.43% and 99.65%, respectively. This article emphasizes that the integration of 3D-printed components has the potential to offset this impact with a substantial 60% reduction. As a result, custom-made solutions involving 3D printing are explored, leading to greater speed, customization, and cost-effectiveness for EVs/AVs. This article also lists the advantages and disadvantages of the existing legislation in Spain, the United Kingdom, and the western Balkans, demonstrating various approaches to promoting electric mobility and the development of autonomous vehicles. In Spain, initiatives like the MOVES program incentivize EV adoption, while the UK focuses on expanding the EV market and addressing concerns about EVs’ quiet operation. In the western Balkans, the adoption of legislation lags behind, with limited incentives and infrastructure for EVs. To boost sales, legal mechanisms are necessary to reduce costs and improve accessibility, in addition to offering subsidies for the purchase of EVs. To this end, an analysis of the incentive measures proposed for the development and use of renewable power sources for the supply of energy for EVs/AVs is presented.

## 1. Introduction

Today’s level of development in science and technology is on the way to enabling the development of a motor vehicle that would satisfy most mobility needs, save energy, protect the environment, and in general meet societal needs with increased automation and, eventually, full autonomy in all conditions of use. It can be said that today marks a major expansion of the automotive industry since the emergence of motor vehicles. Motor vehicle manufacturers are obliged by law to accept responsibility for all phases of the life cycle of motor vehicles (i.e., from their development to the demise of products on the market) in terms of environmental protection and sustainable development. Life cycle assessment is a standardized method to assess environmental impacts [1].

Ultimately, in order to avoid energy and environmental problems today and in the future, new technologies for motor vehicle production focus on the production of automobiles, and they are also facilitated through electric and autonomous vehicles. The deployment of electric vehicles is a potential way to decarbonize road networks, and at the same time, it may offer wider benefits for the environment, such as reducing air pollution and noise in urban areas. A lot of countries have now published their own plans for promoting the uptake of electric vehicles (EVs) and subsequently implemented monetary and non-monetary policies, including subsidies and tax exemptions, for EV owners and deployed charging infrastructure [2]. Also, EVs are gaining momentum due to several factors, including a gradual price reduction, as well as climate and environmental awareness [3]. In particular, to reduce the dependence on oil and environmental pollution, the development of EVs has been accelerated in many countries in an effort to build green cities with low carbon footprints [4,5]. The implementation of new technologies, such as the additive manufacturing of parts (i.e., 3D printing) for EVs/AVs using recyclable materials is also the latest approach in the manufacturing process, from prototyping to even printing entire vehicles, which leads to a long list of benefits, such as vehicle delivery speed, customization, and cost-effectiveness [6].

Today, the EV/AV market is primarily dependent on political support at the national and local levels. This is due to their still-high prices, limited supply of different models compared to conventional vehicles, limited battery charging infrastructure, battery life, driving range, internet development and communication technology, incentives offered for their use, environmental regulations, and fuel economy. Although fully automated vehicles are a matter for the future, the time to regulate them is now. The European Union (EU) aims to create cooperative, connected, and automated mobility based on cooperation between different interconnected types of machinery [7].

In summary, research gaps and perspectives regarding the development of EVs/AVs refer to the context of environmental protection, sustainability, and technological advances. Some research gaps include the need for more studies on the environmental and sustainability impacts of the expanding EV/AV industry. The research prospects include the exploration of new technologies, such as the additive manufacturing of vehicle parts, and their environmental and economic benefits. Furthermore, research is needed to evaluate the impact of government policies, incentives, and regulations on the adoption of EVs/AVs in different regions, as well as the development of cooperative and connected mobility systems, particularly within the EU. Furthermore, continued research is needed to improve the technology, infrastructure, and regulations related to EVs/AVs to ensure their widespread adoption and sustainable development.

### Objectives and Scientific Contribution

The primary focus of this article is to delve into the environmental repercussions of hardware vehicle components and the integration of 3D printing in EVs/AVs, all in the context of sustainability. The article also aims to provide a comprehensive overview of the current state of the automotive industry in terms of sustainability and environmental protection. It discusses the importance of life-cycle assessment as a standardized method to assess environmental impacts and highlights the critical role of well-designed legislation and incentives in overcoming adoption barriers and promoting cleaner and more sustainable EVs/AVs. The article underlines the need for collaboration between all countries in Europe (e.g., EU and non-EU) to address the challenges associated with EVs/AVs holistically. Such collaboration becomes imperative, as a successful transition to greener mobility requires a global effort aligned with sustainability goals. 

In essence, the main research objectives of this manuscript are to identify the factors that affect the carbon footprint of EVs/AVs, to evaluate the potential of additive manufacturing in reducing the environmental impact of the automotive industry, and to discuss the role of regulation and legislation in promoting the adoption of sustainable transportation solutions.

To this end, this paper is organized as follows: Section 1 introduces the context and importance of carbon footprint reduction in the automotive industry. Section 2 explores the relationship between additive manufacturing and sustainability in the production of EVs/AVs. Section 3 analyzes the environmental impact of additional hardware components in EVs/AVs. Section 4 examines the role of legislation and incentives in promoting sustainable transport solutions. Section 5 describes the results obtained from the research and discusses their relevance in the context of sustainability in the automotive industry. Finally, Section 6 presents the conclusions of the study and suggests possible future lines of research.

## 2. Technological Development of EVs and AVs

### 2.1. Electric Vehicles: Past, Present, and Future

EVs are a transport mode that uses an electric motor. That is, EVs are all types of cars that can be powered partly or fully by electricity [2,4,8]. EVs and their application have been actual topics of interest since the appearance of vehicles, but problems with batteries, electric motors, and the conversion of electrical energy that powers those motors, along with cheaper fuel for diesel and gasoline engines that have advanced technically, have led to a situation in which the use and development of electric vehicles have not been of interest for a long time [9].

At the beginning of the 20th century, the popularity of motor vehicles grew rapidly, and the battle for the future of mobility began. During this period, the manufacturers of electric vehicles also worked successfully, but after the first decade of the 20th century and until the oil crisis of 1970, the production of electric vehicles made little progress. After 1970, the most significant shift in the production of vehicles that also have an electric motor alongside their internal combustion engine was made by the Toyota car factory in Japan with its Prius vehicle model. Thus, the Toyota Prius was introduced in Japan (1997) and was the first mass-produced hybrid electric vehicle in the world [10,11].

The renaissance of next-generation EVs began in the 21st century. In the first years of the 21st century, the automobile and electric power company Tesla Motors, based in Palo Alto, CA, USA, the Nissan car factory in Japan, and some Chinese car companies stood out in the production of EVs.

### 2.2. Autonomous Vehicles: Past, Present, and Future

We can say that a vehicle is autonomous if it can perform some human functions as a driver. Fully autonomous vehicles (AVs) can operate without any human control or supervision (i.e., they can perform driving and other activities independently); therefore, we call them driverless/self-driving vehicles [12].

The first AV concept was introduced by the Detroit-based American carmaker General Motors (Detroit, MI, USA) in 1939. The initial phase of research and development was jointly initiated by General Motors and the Radio Corporation of America Sarnoff Laboratory (New York, NY, USA) in the 1950s. In the period from 1964 to 2003, several research and development programs were organized that were operational in the United States, Europe, and Japan as part of individual and joint initiatives of various government institutes and academics to develop automated systems for buses and trucks, super smart vehicle systems, and video processing to recognize driving situations [13]. During the 1960s, Ohio State University developed a driverless vehicle controlled via electronic devices integrated into the road surface, while in the 1970s, the American manufacturing and engineering company Bendix Corporation (Avon, OH, USA) launched and tested the first driverless vehicle, which was controlled using cables integrated into the road surface, with the help of road communicators, for the purpose of receiving and sending digital and analog information to the computer unit in the vehicle.

In the 1980s, the German vehicle manufacturer Mercedes-Benz introduced its version of a robot-controlled vehicle. The vehicle was designed by Ernst Dickmanns and his team from Bundeswehr University Munich in Germany. Following this project, the European Commission started funding the Eureka project “PROMETHEUS—Programme for European Traffic with Highest Efficiency and Unprecedented Safety” on AVs for €749 million in the period from 1986 to 1994 [14].

In the same decade, a group of American universities in cooperation with the USA, the “Defense Advanced Research Projects Agency”, and the American Non-Profit Research Institute developed AVs. At that time, lidar technology was used and tested for the first time (i.e., system management technology using computer vision and robotics). Carnegie Mellon University in the USA has developed a so-called neural network for the control of AVs. In 1994, the German vehicle manufacturer Mercedes-Benz, in cooperation with the Bundeswehr University Munich in Germany, developed a semi-autonomous vehicle [15]. A year later (1995), researchers at Carnegie Mellon University marketed the vehicle with 98.2% autonomy, which was a remarkable step forward in the development of AVs. Using neural network technology, this solution enabled the vehicle to be operated without the use of the hands, but it was still conditioned by human mediation in the process of braking the vehicle. At the end of the last century (1998), the Toyota car factory in Japan offered solutions for speed control systems and maintaining their constancy, and a little later (2009), Google began research on driving a vehicle without the human factor.

**Figure 1:**
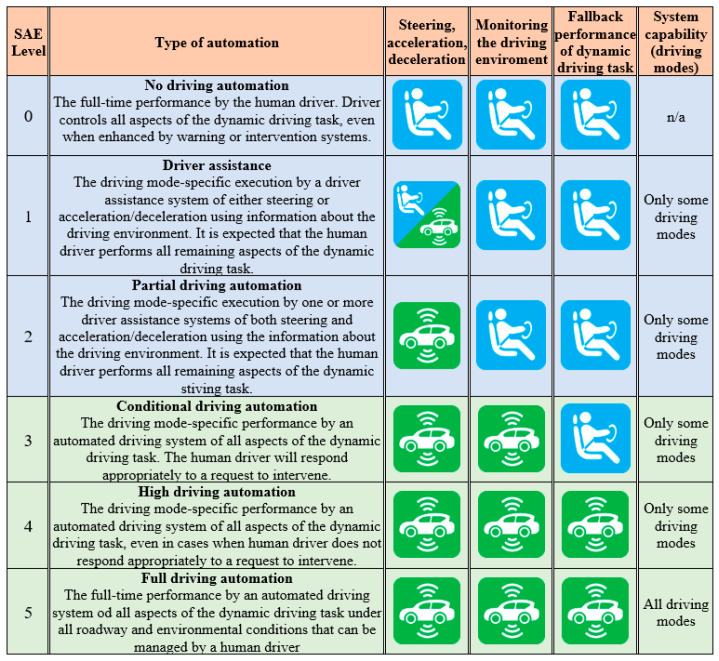
Classification and description of individual levels of autonomy according to the SAE [16]. Blue color means human driver, while green color means automated system.

Today, significant contributions to the development and application of AVs are made by Waymo Google, Uber, Tesla, Renault, Toyota, Audi, Volvo, Mercedes-Benz, General Motors, Nissan, Bosch, and Continental’s autonomous vehicles, etc. The first classification of AVs was developed by the National Traffic Safety Administration—NHTSA (2013)—which defined five levels of autonomy. Also, the Society of Automotive Engineers International—SAE (2014)—published a classification of autonomous vehicles at six levels, called the SAE J3016: JAN2014 standard. This standard was amended in 2016 and was published under the name of the SAE J3016: SEP2016 standard. In 2016, SAE and NHTSA provided an official classification of vehicles according to their degree of autonomy and placed them in six levels. A description of these vehicle autonomy levels is provided in Figure 1.

Therefore, the advancement and rise of autonomous cars have been due to the significant research results obtained in the arenas of wireless and embedded systems, sensors, communication technologies, navigation, data acquisition, and analysis [17]. 

As of 2022, carmakers have reached Level 4. Google’s Waymo partnered with Lyft to offer a fully autonomous commercial ride-sharing service called Waymo One. Projections from manufacturers vary as to when Level 4 and 5 vehicles will be widely available. A successful Level 5 car must be able to react to novel driving situations as well as or better than a human can.

In 2023, intensive work has been taking place on mechanisms to protect AVs from disruptive cyber threats’ key processes, such as decision-making processes or communication processes.

Currently, Level 2 automation is commercially available, and Level 4 automation is only feasible in geofenced locations. Limitations in technology and regulations between countries are significant obstacles to the development of Level 5 vehicles. Developed economies have made more progress in vehicle automation, making them more likely to introduce Level 5 cars first. However, even if a fully developed Level 5 car were available, it could be unusable on most roads due to regulations and infrastructure limitations [18].

### 2.3. Technological Overview of Electric Vehicles

In battery electric vehicles (BEVs), the total electricity is provided by a battery. There is no fuel tank for the storage of fuel, so a BEV is also called a “pure electric vehicle”. It consists of a large, rechargeable battery that does not release harmful toxic gas to the environment, but consumers assume that it creates high pollution levels during the generation of electricity, the manufacturing of batteries, and the dumping of discarded batteries [19].

The performance of a BEV is totally dependent on its battery capacity and its thermal management system. Battery temperature plays a crucial role in governing the performance of the battery and its lifespan. In a BEV, electrical energy is converted to mechanical energy with minimum conversion losses. Vehicle charging times vary with the capacity of the battery, charging scheme, and series/parallel connection used. To increase the distance covered and capabilities, upgraded versions of HEVs, PHEVs, and others have been introduced [20]. 

**Figure 2:**
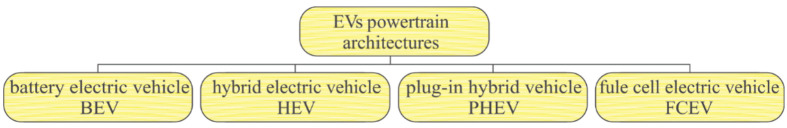
EVs’ powertrain architecture types.

The types of electric vehicles are presented in Figure 2 [4,10].

**Figure 3:**
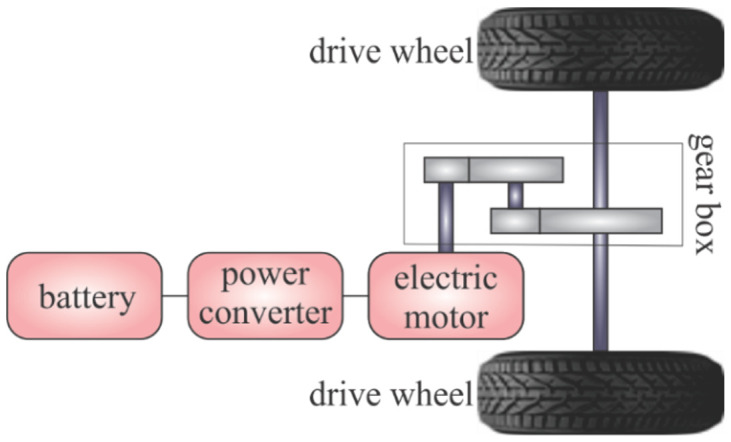
Illustration of a BEV/EV drive.

A BEV is made up of a battery, a power converter, an electric motor, and a gearbox, as Figure 3 shows.

A hybrid electric vehicle (HEV) is a combination of internal combustion engine technology and electric power technology. Depending on the types of energy sources applied to the driveline, an HEV is further classified into three categories: series, parallel, and dual HEVs [21]. A plug-in hybrid vehicle (PHEV) is a combination of battery-powered or petrol-powered vehicles, with the battery being charged at a charging station or at home using an ordinary plug or via a regenerative braking system [20,22]. A fuel cell electric vehicle (FCEV) employs the same propulsion system as a BEV, except that it uses fuel cells to power its battery [23]. In an FCEV, most of the battery is replaced with a hydrogen tank and a set of cells in which the chemical reaction of hydrogen is converted into electricity and water vapor.

The development of battery technology with traction properties in the construction of EVs has had a major impact on the EV industry, as such batteries are used to power EVs’ propulsion systems. Thus, a higher energy capacity, high specific power, high specific energy, and high energy density are required [24]. The most common types of rechargeable batteries used in EVs mainly include lead–acid batteries, nickel–metal hydride (Ni–MH) batteries, and lithium-ion batteries [25]. In addition to battery capacity, charging is another challenge for BEVs.

### 2.4. Technological Overview of Autonomous Vehicles

Vehicle automation and communication technologies are considered promising approaches to improve operational driving behavior [26]. Currently, the AV is undergoing one of its most promising periods in recent years. Areas as diverse as 3D printing, artificial intelligence, laser sensors, cameras, radar, communications, and even the detection and improvement of driver behavior are being developed collectively to improve driving, both in terms of safety and pollution reduction [27].

In simple terms, this means that a vehicle system is composed of various internet and communication technologies such as in-vehicle networking, wireless communications such as 4G/LTE, 5G, 802.11x, and Bluetooth that enable internet access, including cloud and V2X (vehicle-to-everything) communications such as vehicle-to-vehicle (V2V), vehicle-to-pedestrian (V2P), vehicle-to-devices (V2D), vehicle-to-grid (V2G), and vehicle-to-infrastructure (V2I) [28].

**Figure 4:**
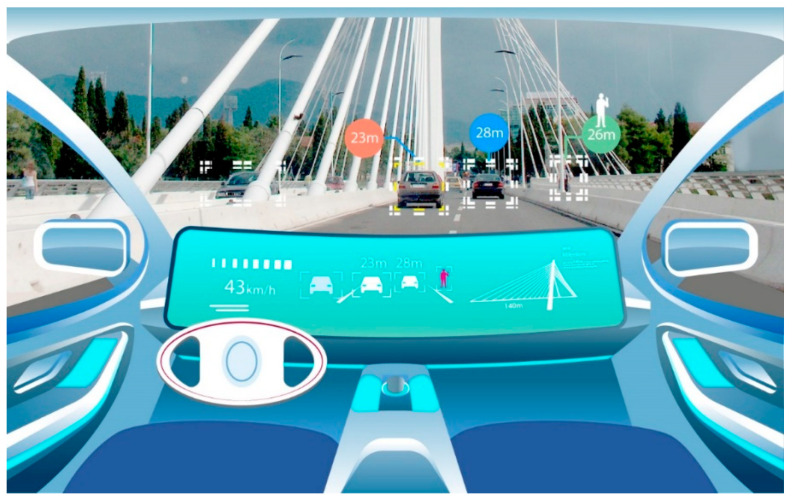
Simulation of AV movement in Podgorica (Montenegro).

Thus, AVs are based on advanced sensors that can collect information about the environment via deep multilayer neural networks, which are used to identify streets, vehicles, objects, and people using data collected from vehicle control sensors. All this is achieved with great computing power, which processes information and converts it into effective commands in real time. The sensor sets on AVs (cameras, lidars, radars, etc.) complement each other and compensate for any weaknesses of any sensor used, as Figure 4 shows.

The architecture of AV systems is presented from a technical perspective, which includes hardware and software components, and from a functional perspective, which describes the processing blocks required within the autonomous vehicle itself, from data collection to vehicle control. AV systems consist of four primary functional blocks, namely [29]:

- Environment perception and modeling;
- Map positioning and construction;
- Vehicle path and movement planning and control;
- System monitoring.

The sensor system consists of several different sensors that have the task of collecting data from the vehicle’s environment in real time. The data collected via the sensors are used for perception, route planning, obstacle distance calculation, or navigation. AVs have short, medium, and long-range sensors such as ultrasonic sensors, capacitive sensors, infrared sensors, lidar, radar, global positioning systems (GPSs), etc. [30,31,32,33,34,35].

The development of AVs is a rapidly growing field that holds great promise for the future of transportation. However, navigating these vehicles in complex and unconventional scenarios is still a major challenge. The most difficult scenarios for AV algorithms, as well as human drivers, to react to were found to be harsh weather conditions, unsignalized intersections, traversing crosswalks, navigating roundabouts, and near-accident situations [36].

The primary objective of autonomous cars is to mitigate accidents and human errors, thus enhancing road safety. However, several unresolved challenges persist in developing AVs. The software and system requirements are among the aspects that demand consideration in vehicle creation. While these aspects hold minimal significance in traditional vehicles, self-driving cars can potentially trigger damage, accidents, and diminished safety. Numerous challenges have arisen in this realm, primarily stemming from the intricacies of system design and the critical demands of data collection at the network level, which subsequently inform decision-making processes [37].

Automotive manufacturers and policymakers seek new solutions in managing data and connectivity, ensuring robust infrastructure to support AVs, vehicle updates and maintenance, and post-production and post-deployment services. The concerns and growing challenges remain unabated, and global societal voices call for industry engagement and collaboration on new technologies and strategies, as well as understanding the impacts of the transformation to mobility-as-a-service (MaaS) and robotaxi services [38].

### 2.5. Differences between EVs/AVs and Conventional Vehicles

EVs/AVs offer several advantages over conventional vehicles (CVs) that use petrol or diesel, especially when it comes to emissions, noise emissions, non-renewable energy consumption, and maintenance costs. Nevertheless, intelligent vehicles are equipped with advanced sensors, controllers, and actuators in combination with connecting communication technologies compared with CVs, for which the energy will definitely increase [39]. On the contrary, EVs/AVs cause zero emissions during operation. Certainly, when it comes to the stopping phase, they also emit particles from tires and the friction elements of the braking system.

The production of EVs’/AVs’ batteries and their subsequent disposal after decommissioning pollute the environment, but this pollution is easier to control in relation to the emission of harmful substances from CVs. The complete supply chain of CVs with petrol or diesel pollutes the environment.

EVs’/AVs‘ and CVs’ powertrains are fundamentally different, and due to their external characteristics (i.e., depending on the torque and/or power of the output shaft on the number of revolutions), electric motors are more practical to use. This results in EVs/AVs‘ utilizing over 85% of the energy invested in their operation.

To start CVs, it is necessary to ensure the start of the internal combustion engine with a low speed and low torque on the engine’s flywheel. Unlike CVs, high torque can be transmitted to the motor shaft at start-up for EVs/AVs. Because of this, EVs/AVs do not need a mechanical power transmission to change gears.

EVs/AVs in the braking phase can accumulate (recover) energy. The recovery of energy is especially noticeable when moving on sections of the road that are falling and the brake is often used, and also in city driving, because the start-stop phases are often changed.

CVs are more convenient to use than EVs/AVs when it comes to the radius of movement. EVs/AVs still have a limited range and depend primarily on battery capacity. Due to the large presence of electronics within the vehicle, EVs/AVs have significantly higher energy consumption compared to CVs [40]. With the level of vehicle autonomy, the carbon footprint increases [39].

### 2.6. Carbon Footprint of the Additional Hardware of EVs and AVs

The comfort and safety elements of classic vehicles have a great impact on carbon footprints and, therefore, on the economy. To measure their implication, it must be considered that 100 watts typically corresponds to fuel consumption of 0.1 L every 100 km. For a journey time of 1 h, the power consumption is equivalent to 100 Wh, and the carbon footprint (carbon footprint vs. power consumption, available at www.ceroco2.org/calculadoras/ (accessed on 7 November 2023)) is 0.03 kg of CO_2_ emitted into the atmosphere. Taking as a reference the annual average price of CO_2_ emission rights according to the European Trading Scheme (European CO_2_ trading system, available at www.sendeco2.com/es/ (accessed on 7 November 2023)) (ETS), this equates to a monetary cost of ~2.44€ (81.34€, according to the spot price calculated on 15 September 2023 using EU allocations). In this sense, Table 1 shows the carbon footprint of some typical components aboard traditional vehicles.

Table 2 shows the battery packs used in current EVs and the impact of power consumption on autonomy and carbon footprints. Note that CO_2_ emissions are measured in grams per every 100 km traveled, according to the EU Energy Label.

Modern EV/CVs are considered networks on wheels—in practice—as they are more connected to the internet, whose evolution of AVs will make them true data centers on the road [39]. While PC gaming and smartphones currently increase RAM memory by up to 16 GB and 18 GB, respectively, the resource requirements of vehicles to meet Level 3 driving capabilities in 2022 were expected to reach 140 GB of RAM and 1 TB of internal storage. The consequence was that advanced driver assistance systems (ADAS) consume a considerable amount of energy and put a strain on batteries. 

For further analysis, Table 3 shows the carbon footprint of some typical components aboard modern vehicles [41]. The latest cars equipped with cameras and radars typically generate ~12 GB of data every minute. Processing these images requires a lot of computing power, whose consumption can reach around 2500 watts in some prototypes, enough to power 40 incandescent light bulbs. From Table 4, this results in 750 g of CO_2_ emissions with a cost of 61.00€ every 100 km. As another example, the GM Cruise AV is equipped with 5 lidars, 16 cameras, and 21 radars. This results in ~7200 watts or 2.15 Kg of CO_2_ emissions with a cost of 174.88€ every 100 km.

According to a study [41], it can be inferred that an average intelligence level dramatically increases the carbon footprint to 468 g of CO_2_ every 100 km (i.e., 38.07€). Similarly, the carbon footprint of a basic intelligence car increases by 780 watts/100 km compared with classic vehicles, whilst the footprint of an advanced intelligence car increases by 1860 watts/100 km. In sum, the carbon footprint increases as the autonomous driving capability increases, with the automation function being the main cause and the connection function being the second one (Table 4).

Hopefully, the future will be promising, and manufacturers such as, e.g., Nvidia, Intel, Qualcomm, and Tesla, are directing their efforts to decrease the size of in-vehicle electronics and reduce massive electrical consumption by developing low-power chips optimized for AVs. For example, Nvidia has designed a new processor called Xavier based on an octa-core CPU, a 512-core GPU, a deep learning accelerator, computer vision accelerators, and 8 K video processors with just 30 watts. Thus, fully autonomous driving could be a reality with only two processors and two dedicated GPUs—to ensure vehicle safety through the redundancy of the computing platform—with an acceptable power consumption of 500 watts and 150 g of CO_2_ emissions every 100 km [40].

Moreover, there is also a significant demand for solutions to move the intelligence aboard vehicles to remote intelligence in cloud computing systems. Remote intelligence will increase the management of AVs and their systems, increase computing capacity without requiring excessive hardware growth in cars, dramatically reduce the cost of on-board electronics and their carbon footprint, as well as allow greater synergy between the system control functions and online services. Addressing latency challenges is among the current priorities of both researchers and manufacturers. To this end, manufacturers are currently investigating distributed platforms for automotive services with real-time cloud access, thus making vehicles part of an intelligent transportation system (ITS) [42]. This is the case of REMOTIS, a remote intelligence system for cars capable of connecting to a server and transferring all the information from their sensors to carry out critical driving functions remotely through a 5G infrastructure (https://www.youtube.com/watch?v=uQk1kljyXZ4 (accessed on 7 November 2023)).

### 2.7. Implementation of Additive Manufacturing in EVs/AVs

A very important part of creating harmony between the accelerated technological development of EVs/AVs and the environment is the implementation of additive manufacturing (AM) and the usage of recyclable materials in 3D-printed automotive parts. AM, popularly called 3D printing, is the process of building a physical object using modeling data, and it represents one of the most revolutionary technologies of this era. 

The first idea connected to creating this technology came from the last century and was based on the simultaneous photography of an object from different angles using 24 cameras while making a 3D model [43]. This technology has facilitated the classical mass production of 3D-printed items and parts, a process that encompasses product design, involving iterative collaboration between production engineers and designers, optimization, production analysis, printing modules, post-production, and more [44]. The benefit of additive manufacturing compared to classical manufacturing is that it applies a trial-and-error technique, which allows a designer to print a model and use it in practice without suffering any losses (other than filament losses). If a model does not operate as expected, the designer can make any necessary corrections until it performs all the desired functions.

The most important implementation of 3D printing has been in the areas of automotive manufacturing, medicine, architecture, art, design, and lately, even food [45]. Moreover, due to advances in 3D printer technology, the market for 3D printing has recently experienced some of the manufacturing sector’s fastest growth. Especially after the outbreak of the COVID-19 pandemic, the 3D printing of protective equipment (e.g., face shields, masks, and bracelets) was essential for the whole world, and both freelancers and academics did their best to design such equipment [46,47]. 

In 2009, the company MakerBot produced so-called “desktop” 3D printers, which were used for people to perform 3D modeling and printing at home [48]. Today, MakerBot’s printer is sold as a kit that users assemble into a finished printer. The MakerBot company also created the first online library (i.e., Thingiverse) where files that can be 3D-printed and downloaded for EVs/AVs can also be found, and which is becoming the largest online community in the world concerning 3D printing [49].

Today, there are several types of 3D printing machines or techniques, depending on the size, complexity, and scale of the product. The way an object is produced differs, depending on the type of printer used. There are many types of 3D printers, and their classification can be achieved according to several criteria: technology, materials, and purposes [50]. To produce EVs/AVs, industrial 3D printers are mainly used and specifically designed for these applications. While price is an important factor, the real key differentiators are the specifications, performance, features, and functions offered by each industrial 3D printer, such as the following: the ability to work with materials of high performance and engineering quality; the possession of a large, actively heated chamber for building objects; a high printing speed, which equals high productivity; precision and the accuracy of dimensions; repeatability and reproducibility; operator safety, monitoring and productivity; and an open platform with certified materials.

According to a report from 2017 with the greatest emphasis on the analysis of countries in Europe and their users, the most commonly used materials for 3D printing are still polymer-based (plastic materials)—as much as 88% [51]. Resin is the second-most-used material with characteristics of high resistance and strength. Of the metal materials that are in third place, both pure metal powders and alloys are used, most often stainless steel and alloys of aluminum, chromium, cobalt, nickel, etc. Apart from the mentioned materials, various types of ceramic powders based on zinc or aluminum can be used, then come powders based on gypsum, cellulose, various types of sand powders, biocompatible powders, etc. Precisely in the last few years has the increasing consumption of these materials has been observed [52].

The automotive industry is the fastest-growing vertical using AM; therefore, the EV/AV market represents a great fit for AM as a production process. Many companies, instead of traditional plastic-injection-molding processes, choose cost-effective 3D printing processes, especially if they need fewer than 50,000 parts a year. Nowadays, manufacturers face low production volumes and highly uncertain demand. That is why automakers ranging from Ford and Volkswagen, along with startups such as Arash Motor Co. and Rivian, are investing highly in AM technology. The adoption of 3D printing in EV/AV manufacturing processes offers a multifaceted approach to environmental sustainability, including circular economy principles and energy optimization. For instance, 3D printing has potential in the circular economy of plastic components at the end of the lives of vehicles, offering opportunities for recycling and the decentralization of supply chains [53]. Additionally, 3D printing has demonstrated substantial energy savings through selective laser sintering (SLS) in the automotive and aircraft industries [54]. Other factors attributable to 3D printing in the broader industry (e.g., material efficiency, lightweighting, and customization or localized production) may also contribute to reducing the carbon footprint associated with the manufacturing processes of EVs/AVs, fostering environmental sustainability while enhancing economic viability. 

On average, EVs/AVs could incorporate between 50 and 200 3D-printed parts (e.g., interior components, support parts, and prototype/tests, as well as custom components for sensor systems and autonomous technology). When the average values from Table 5 are employed, the cumulative effect suggests an overall conservative reduction of approximately 60% in the carbon footprint. This assessment serves as a simplified estimation, and real-world impacts may significantly fluctuate, contingent upon the circumstances of each production process and vehicle model (i.e., more advanced and customized vehicles may have a greater number of 3D-printed parts, while more conventional models may have fewer). Furthermore, continued advancements in technology and materials hold the potential to further amplify these benefits over time.

## 3. Regulation and Legislation

A few countries are at the forefront of regulation in this fast-growing field. The country selection in this article followed a series of discussions among the team and was also based on the outputs of the WISE-ACT (https://www.wise-act.eu (accessed on 7 November 2023)) network [55]. Legislation and deployment approaches naturally differ worldwide. Yet, the EU has been a pioneer in starting the discussion about AV/EVs, particularly regarding legislation and support, such as in the Green Deal [56]. Additionally, the EU has attempted to follow an individual path, differing from the approaches adopted by both China and the United States regarding the deployment of AVs and EVs. This approach was founded on policy development within the EU, particularly focusing on data management, which is a core element of, e.g., DSA, GDPR, and MDMS.

Nonetheless, the EU includes 27 individual countries, so it is also interesting to highlight diversity. Spain has been among the last EU countries to comply with AV legislation requirements [57] despite the 2015 DGT allowing AV testing, but it has been making forward leaps since 2022, as outlined in Section 4.1. The UK was an EU member until 2020 but has followed an individual path since its aim has been to be a global leader in this field. The establishment of the CCAV (the Centre for Connected and Autonomous Vehicles) at the national level and the introduction of relevant legislation via the Law Commission (https://lawcom.gov.uk/project/automated-vehicles/ (accessed on 7 November 2023)) have contributed to the UK being at the forefront of this field. On the other hand, the western Balkans are developing their own relevant infrastructure and are working to integrate their policies with existing EU policies. To date, less attention has been paid to countries with lower technological development levels, and hardly any AV trials have taken place, so a perspective such as the one offered by this article adds valuable insight for both practitioners and policymakers.

Consequently, focusing on the EU at a supranational level and on Spain, the UK, and the western Balkans at a national level provides valuable insight into developments and best practices in this field. The fact that major automotive manufacturers are based in Spain, the UK, and the EU as a whole (e.g., Nissan, Tesla, Jaguar, or Polestar) further justifies the selection of these countries as examples to draw useful teachings for other countries across the globe when focusing on these technologies.

### 3.1. Advantages and Disadvantages of Existing Legislation for Spain, UK and at the EU Level

Despite the numerous advantages of EVs, the probability of being run over when cars only operate with electrical mechanics increases by 40%, according to studies in Spain. Also, the NHTSA concluded that such cars are 19% more likely to cause accidents [58]. The reason is that electric drive motors emit little noise at low speeds, making EVs potentially dangerous for pedestrians and cyclists. To reduce accidents, all new EVs, hybrids, and plug-in hybrids sold in Europe since 2021 are equipped with an acoustic vehicle alerting system (AVAS). Regulation No. 138 of the United Nations Economic Commission for Europe (UNECE) was already mandatory in 2019 for newly approved cars. This update is very good news in terms of safety, as regulation will help reduce deaths or injuries. However, it may not contribute to noise pollution reduction in cities. For instance, the World Health Organization (WHO) recommends maximums of 53 dB and 45 dB for traffic noise at day and night, respectively. This exceeds the values between 56 and 75 dB set by Europe for AVASs.

Regarding AVs, the objective of the EU is to achieve zero victims on the road by 2050. These plans are going through what institutions have called Vision Zero, a project that aims to save 25,000 lives and prevent 140,000 serious injuries. For this, eight ADASs will be mandatory in homologated cars and for sale in Europe from 2022 and 2024, respectively. They include an intelligent speed assistant (ISA), a fatigue and drowsiness detector, emergency braking, a rear camera with a cross-traffic alert, a lane change alert, a seat belt alert in the rear seats, a black box, and an integrated breathalyzer. To this end, legislation still needs to be further developed to be in tune with ICTs. In this manner, the official traffic code must be modified to establish rules consistent with the philosophy of autonomous cars. Also, the Law on Civil Liability and Insurance must confirm the responsibility of drivers regarding damages caused to people or properties due to autonomous driving [59].

### 3.2. Advantages and Disadvantages of Existing Legislation for the Western Balkans

The rapid technological development of EVs/AVs has led to a backlog of legislation governing their use on the road. However, the basic precondition for making transport systems safer and more efficient is the establishment of harmony between the technical solutions of vehicles and the regulations for their use.

Regardless of the economic development of certain countries in the western Balkans (WBs), there is already a need to have legislation adopted at the national level that contains regulations for the use of Level 5 AVs (i.e., fully autonomous vehicles). The key reason for the delay in the adoption of legislation at the national level for the use of Level 5 AVs in the WB countries is their availability to the market and customers, especially in the years of the global health crisis caused by the COVID-19 pandemic and its negative impact on the automotive industry.

Only the WB countries that are members of the EU have adjusted their national regulations for the use of EVs in terms of rights and obligations for the use of cleaner and energy-efficient vehicles in traffic. Also, in a white paper (2011), the EC adopted a plan for a single transport market with competitive transport systems and the efficient use of resources. The document states that new technologies for vehicles and traffic management will be key to reducing transport emissions in the EU and the rest of the world. One of the goals defined in the document is to halve the use of conventional vehicles with fossil fuels in urban areas by 2030 and to completely ban their use in urban areas by 2050. This indicates that the WB countries need to work intensively to enact legislation that will implement the EC recommendations given through the content of the white paper or the EU Directive.

Legislation in the field of electromobility in the EU (e.g., regulations 1316/2013, 2009/28, 2009/33, 2014/94, 2018/2089 (INI), or 2019/2144) should be implemented in WB countries to decarbonize transport as soon as possible with the elimination of obstacles related to the infrastructure of charging stations and informing citizens about the benefits of EVs/AVs (https://www.surrey.ac.uk/research-projects/co-creating-accessible-futures-through-new-mobility-services (accessed on 7 November 2023)). With the implementation of regulation (EU) 2019/2144 for the first time in 2019, the definition of an automated vehicle in the part concerning compliance with homologation regulations for vehicles has also appeared in the WB countries. Automated vehicles have the potential to make a huge contribution to reducing road fatalities, given that more than 90% of road accidents are estimated to result from some level of human error. As automated vehicles gradually take over a driver’s tasks, harmonized standards and technical requirements for them should be adopted at the EU level (including a verifiable safety guarantee for decision-making by automated vehicles). All this must be promoted internationally within the framework of the UNECE’s World Forum for Harmonization of Vehicle Regulations (Article 29 Working Party) while respecting the principle of technological neutrality [60].

It is imperative that the WB legislation does not contain barriers to the import of EVs/AVs. Also, it can be stated that in the WB countries, through the National Strategies of Sustainable Development, Transport or Energy, this importance is recognized, but small steps are being taken to establish an efficient electromobility system addressing governmental, industrial, and wider societal goals. We singled out the WB countries that have a national legislative framework through which certain incentive measures and facilities for the purchase of EVs are defined.

- Montenegro: the Law on Environment; the Statute of the Environmental Protection Fund; the ordinance on the conditions that must be met by users, the manner of allocating and using the funds of the Environmental Protection Fund, and the Law on Tax on the Use of Passenger Motor Vehicles, Vessels and Aircraft;
- Serbia: the Law on Environmental Protection and decree on the conditions and manner of conducting subsidized purchases of new vehicles that have an exclusively electric drive, as well as vehicles that have an electric drive with an internal combustion engine;
- Northern Macedonia: the Law on Motor Vehicles and the decree on the method of calculating the tax on motor vehicles;
- Bosnia and Herzegovina: no lists would explicitly legally regulate the facilities for the use of EVs.

The situation elsewhere in the region is much better; e.g., Slovenia and Croatia have implemented all EU regulations in their national legislative frameworks. Their national frameworks contain the following.

- Slovenia: the Law on Environmental Protection; the Energy Law; the Act on the Establishment of the Public Fund for Environmental Protection; the general business conditions of the Public Fund for Environmental Protection; and the Program of the Public Fund for Environmental Protection;
- Croatia: the Law on the Fund for Environmental Protection and Energy Efficiency; the Statute of the Fund for Environmental Protection and Energy Efficiency; the ordinance on the conditions and manner of allocating funds from the Fund for Environmental Protection and Energy Efficiency, as well as criteria for assessing applications for allocating funds from the fund; the ordinance on the manner of monitoring the intended use of the Fund for Environmental Protection and Energy Efficiency and contracted rights and obligations; the ordinance on the procedure for announcing tenders and on deciding on the selection of beneficiaries of the Fund for Environmental Protection and Energy Efficiency; and the Energy Efficiency Act.

Overall, however, the aforementioned national legislation could be further improved to support electromobility in some countries. 

## 4. Analysis of Existing Encouraging Measures for Putting EVs and AVs into Use

### 4.1. Analysis at the EU Level

In Spain, electric mobility and the deployment of recharging infrastructure for these vehicles have been promoted annually since 2019 via MOVES (i.e., a Spanish acronym for the Program of Incentives for Efficient and Sustainable Mobility). This aid program subsidizes the purchase of electric, plug-in hybrid, or fuel-cell vehicles for large companies, SMEs, or individuals with an endowment of up to €15,000. In 2021, the goal was to give a significant boost to the automotive sector with a budget of 400 M€—expandable to 800 M€—and achieve 250,000 EVs and 100,000 charging points by 2023 [61].

Regarding AVs, Spain took the first step in 2015 when the General Directorate of Traffic (DGT) issued an instruction to allow tests with Level 3, Level 4, and Level 5 AVs on roads with traffic. More recently, Spain and Portugal announced in 2018 another initiative aimed at putting into operation two road corridors—of 153 km and 163 km—with access to 5G networks to test AVs. Although this instruction has facilitated tests to achieve full autonomous driving, the 2015 DGT guideline is very limited. Therefore, the new Road Safety Law introduced an amendment in 2022 intending to regulate the obligations, use, and development of AVs. For this purpose, carmakers must communicate with the Vehicle Registry of the Central Traffic Headquarters the capabilities of the automated driving system at the time of the vehicle registration, as well as any wireless updates of the system throughout its useful life. As a result, this law is expected to boost the development and commercialization of Level 3 AVs in Spain [62].

On the contrary, regulations have been continuously developing in the UK. Such developments have been supported by, for instance, CCAV and the UK Law Commission, as mentioned in Section 3. However, the 2023 announcement by the UK Government about prolonging green targets threatens AV/EV deployment and affects the long-term investment plans of automotive manufacturers.

### 4.2. Analysis in the Western Balkans

In general, when it comes to the buyers of vehicles from the WB countries, there is less motivation for them to buy EVs/AVs. The main reasons for their lower motivation to buy EVs/AVs are their high purchase price, the low number of vehicle models, limitations on battery charging and total infrastructure for their use, their limited/small radius of movement, the lack of information about their capabilities, and political/incentive measures concerning their acceptance.

The market analysis conducted within this study about WB countries concluded that there is no well-developed network of authorized dealers and distributors offering EV models to consumers. Thus, there is an insufficient availability of EVs, services, commercial financing, and the required number of filling stations. What would be necessary to achieve more significant sales of EVs in the WB countries would be to define legal mechanisms to reduce purchase costs (tax exemptions), provide public infrastructure for charging batteries, reduce fees for engaging network capacity when charging batteries, reduce purchasing prices through subsidies, introduce ecological zones, reduce parking fees, reduce road maintenance fees, etc.

Today, of the WB countries, only Bosnia and Herzegovina and Kosovo do not have funds to subsidize the purchase of EVs. The countries of the WB and the regions that have provided subsidies for the purchase of EVs in 2021, as well as their amounts, are as follows.

- Montenegro: an incentive for the purchase of electric and hybrid vehicles; a total of €100,000 was earmarked for this purpose (2021), of which €50,000 was marked for electric vehicles and €50,000 for hybrid vehicles; €5000 per individual for M1 electric vehicles, €2500 for M1 hybrid electric plug-in vehicles with CO_2_ emissions of less than 50 CO_2_ (g/km), and €2500 for M1 full hybrid vehicles with CO_2_ emissions up to a maximum 130 CO_2_ (g/km);
- Serbia: €5000 individually for M1 and N1 vehicles powered exclusively using electricity and €3500 for M1 and N1 vehicles that are hybrid plug-ins, as well as vehicles that are range-extended, with CO_2_ emissions/km up to a maximum of 50 g/km;
- Northern Macedonia: the owners of electric vehicles are exempt from paying the motor vehicle tax, while for hybrid plug-in vehicles, 50% of this tax is paid.

Also, some countries of the region, Slovenia and Croatia, offer subsidies for the purchase of electric and hybrid vehicles that are an order of magnitude higher than those of, for example, Montenegro and Serbia.

The investment in EVs is worthwhile, based on this article’s analysis. This is particularly true when EVs cover a significant number of kilometers annually, especially when incentives of €5000 or more for their purchase are available.

## 5. Discussion

The review conducted in this article revealed several key aspects related to sustainability, carbon footprints, 3D printing, regulation, and legislation in the context of EVs/AVs and their adoption in Europe and western Balkan countries.

### 5.1. Discussion on Sustainability, Carbon Footprint, and 3D Printing

In the context of sustainability, the findings underscore the challenges and opportunities associated with the adoption of EVs/AVs. While EVs are recognized for their potential to reduce emissions and dependence on fossil fuels, the current analysis emphasizes that their environmental benefits are contingent on various factors. For example, the reduction in emissions depends on the source of electricity generation used to charge EVs. The manufacturing process of EV batteries and their disposal at the end of the life cycle can also have environmental implications. Notably, EVs utilize over 85% of the energy invested in their operation compared to CVs, which have a lower energy efficiency rate. This highlights the trade-off between automation and the environmental impact, as AVs tend to have higher energy consumption.

Additionally, the analysis indicates that the carbon footprint of AVs is influenced by ADASs and the computational resources required for autonomous driving. The development and deployment of AVs and their associated technologies, such as sensors, cameras, and processing units, can lead to increased energy consumption and carbon emissions. For instance, some advanced AVs equipped with multiple sensors, cameras, and processors can generate approximately 12 GB of data every minute, requiring substantial computing power. This heightened energy consumption can result in emissions equivalent to powering 40 incandescent light bulbs or 2.15 Kg of CO_2_ emissions every 100 km. Likewise, the energy consumption of advanced-intelligence cars can increase by 1860 watts/100 km compared to CVs, highlighting the trade-off between automation and the environmental impact. These findings underscore the importance of considering the environmental implications of both the hardware and software components of AVs, as well as the manufacturing methods employed. In summary, these results highlight the need for a holistic approach to assess and mitigate the environmental impacts of EVs/AVs, considering factors such as energy sources, battery production, disposal, and sustainable manufacturing practices like 3D printing.

This article underscores the significance of 3D printing in the manufacturing of EVs/AVs, particularly in the context of material efficiency, lightweighting, customization, local production, recycling, circular economy, and energy efficiency. It highlights that AM technologies offer a sustainable approach by enabling the production of automotive parts using recyclable materials. The use of industrial 3D printers specifically designed for automotive applications is on the rise, bringing benefits such as reduced waste, cost-effectiveness, and customization. Numerical data indicates that industrial 3D printers tailored for automotive applications provide cost-effective and environmentally friendly solutions, especially for production volumes below 50,000 parts annually. Therefore, industry leaders like Ford and Volkswagen are making substantial investments in AM technology, reflecting the automotive sector’s commitment to sustainable practices. The integration of 3D printing into the manufacturing process aligns with the broader sustainability objectives of reducing environmental impacts and optimizing resource utilization in the automotive industry. The cumulative effect of factors in the 3D printing of EVs/AVs suggests a potential overall reduction of approximately 60% in carbon footprints.

### 5.2. Discussion of Regulation and Legislation

This article provides insights into the challenges faced by both more and less advanced economies in promoting the adoption of EVs/AVs. This review has highlighted the role of legal mechanisms and incentives in shaping consumer behavior and industry development. It has emphasized that high purchase prices, limited model availability, and inadequate charging infrastructure act as barriers to adoption, a finding consistent with previous studies on EV market dynamics. 

Furthermore, this manuscript has discussed the importance of introducing supportive policies and incentives, such as tax exemptions, public charging infrastructure, and subsidies, to stimulate EV adoption. For instance, Spain’s MOVES program, with a budget of up to €800 million, aims to achieve 250,000 EVs and 100,000 charging points by 2023. This illustrates the potential effectiveness of such initiatives in driving EV adoption and reducing carbon emissions from the transportation sector. However, this analysis has also revealed disparities among western European countries and WB countries in terms of their readiness to adopt and promote EVs/AVs. For example, some European countries have implemented EU regulations in their national legislative frameworks, while others are still undergoing this process. The varying levels of subsidies and legislative frameworks across these countries underscore the need for consistent and coordinated efforts to accelerate sustainable transportation transitions in the region. These findings align with previous research on the role of government policies and incentives in shaping the electric and autonomous vehicle markets.

To sum up, the analysis presented in this work emphasizes the multifaceted nature of sustainability and regulatory challenges in the context of EVs/AVs, not only in WB countries but also in western European economies. It underscores the importance of considering the entire life cycle of these vehicles, from production to disposal, to accurately assess their environmental impacts. Additionally, the findings highlight the critical role of well-designed legislation and incentives in overcoming adoption barriers and promoting cleaner and more sustainable transportation options. To successfully transition to a greener mobility future, both western European economies and WB countries need to address these challenges comprehensively and collaborate with international partners to align their strategies with global sustainability goals.

## 6. Conclusions

This review underscores the substantial impact of comfort and safety features in CVs on carbon footprints and their subsequent economic implications. Furthermore, it highlights that intelligence levels and autonomous driving capabilities significantly contribute to increased carbon emissions, with automation functions and connectivity features playing pivotal roles. While EVs have increased their carbon footprint by 12.13% compared to conventional vehicles, AVs with basic and advanced intelligence experience have increased theirs by 41.43% and 99.65%, respectively. To address these concerns, manufacturers are actively pursuing the miniaturization of electronics and the development of low-power chips optimized for AVs. The integration of innovative manufacturing methods such as 3D printing is gaining momentum in automotive production, offering the potential to reduce both material waste and carbon emissions. In this sense, the integration of 3D-printed components has the potential to offset this impact with a substantial 60% reduction. Moreover, there is a growing demand for shifting intelligence from on-board vehicle systems to remote cloud computing, a transition that promises the enhanced management of AVs, increased computing capacity, reduced on-board electronics costs, lower carbon footprints, and improved synergy between system control functions and online services.

In recent years, the technology and legislation related to EVs/AVs have significantly evolved. This progress necessitates state-of-the-art supporting infrastructure for safer operations, including ADASs and super-fast 5G wireless technologies for Level 5 vehicles. This is particularly relevant due to a 40% increase in the likelihood of pedestrians being struck by EVs in Spain and a 19% higher propensity for EVs to cause accidents, as reported by the NHTSA. In this context, the EU has set ambitious targets for AVs, aiming to achieve zero traffic-related deaths by 2050. This goal is based on the implementation of ADASs in vehicles, which is expected to save approximately 25,000 lives and prevent 140,000 serious injuries. These innovations in EVs/AVs underscore the critical need to prioritize safety, the imperative to harmonize legislative frameworks in Europe, including the western Balkans, and the proactive measures aimed at driving the adoption of EVs/AVs in the region.

From this perspective, this article has discussed the advantages and disadvantages of existing legislation at the national, regional, and EU levels, considering the dynamics of the global marketplace in which electric and autonomous transportation operate freely. It has underscored the importance of developing a supporting industry and the necessary infrastructure, including 3D printing facilities, to accommodate the growing mass adoption of electric and autonomous mobility. Finally, this review has presented a comprehensive analysis of incentive measures aimed at promoting the development and utilization of renewable energy sources for powering EVs/AVs. 

Future research avenues include exploring advanced battery technologies with improved energy density and sustainability. Investigating methods to reduce the energy consumption associated with autonomous driving systems, such as more efficient algorithms or low-power computing solutions, will be crucial. Further studies should also delve into the environmental impact of 3D printing in AM, with a focus on optimizing materials and processes for greater sustainability. Additionally, research on the development and implementation of harmonized regulations and incentives for EVs/AVs and their data management across different regions and countries will be essential to promote widespread adoption and address carbon emissions effectively. Finally, assessing the long-term social and economic impacts of transitioning to electric and autonomous mobility systems should be a priority to ensure a holistic understanding of the implications of these technologies on society and industry.

## Figures and Tables

**Table 1:** Carbon footprints of some typical components aboard classic vehicles.

Component	Consumption (Wh)	Carbon Footprint (CO_2_ g/km)	Cost (€)
Heated windscreens	120	32	2.60
Windscreen wipers	80–150	20–40	1.63–3.25
LED lights	50	10	0.81
Heater	170	40	3.25
Seat heaters	100–200	30–50	2.44–4.07
Air conditioning	500	130	10.57
Sunroof	200	50	4.07
Power windows	150	40	3.25
Plug socket	50	10	0.81
Radiator fan	800	200	16.26
Engine controller	200	50	4.07
Headlight cleaning	100	30	2.44

**Table 2:** Carbon footprints of the current main EVs.

Vehicle	Battery Pack	Total Energy (kWh)	Consumption (kWh/100 km)	Autonomy (h)	Carbon Footprint (CO_2_ g/km)	Cost (€)
BMW i3	96 Li-ion cells	22	12.34	1.78	59	4.80
Nissan LEAF	192 Li-ion cells	24	17.2	1.4	54	4.40
Volvo C30	384 Li-ion cells	24	9.47	2.53	120	9.76
Mini Cooper SE	192 Li-ion cells	32.6	15.5	2.10	45	3.65
Mercedes B-Class	3696 NCA cells	36	16.6	2.17	146	11.88
Toyota RAV4	4500 Li-ion cells	41.8	16.6	2.52	103	8.37
Renault Zoe	192 Li-ion cells	52	17.7	2.94	43.88	3.56
BYD E6	LiFePO4 cells	61.4	19.5	3.15	N/A	N/A
Hyundai Ioniq 5	384 Li-ion cells	72.6	16.8	4.32	63	5.13
Kia EV6	384 Li-ion cells	72.6	20	3.63	63	5.13
Mercedes EQC	384 Li-ion cells	70	22.2	3.15	25.5	2.07
Tesla Model S	7104 Li-ion cells	85	18.1	4.59	26	2.12

**Table 3:** Carbon footprints of some typical components aboard modern vehicles.

Component	Consumption (Wh)	Carbon Footprint (CO_2_ g/km)	Cost (€)
Camera	3	0.9	0.07
Radar(middle distance)	4.5	1.35	0.11
Radar(long distance)	6.25	1.875	0.15
Lidar (32 lines)	12.1	3.63	0.29
Lidar (64 lines)	60	18	1.46
Ultrasonic sensor	0.13	0.039	0.003
GNSS positioning and inertial navigation	3.9	1.17	0.09
Processor	500	150	12.20
V2X chip	6	1.8	0.15
MIMO RF module	1	0.3	0.024
Gateway	36	10.8	0.88
Display	60	18	1.46
Head-up display	8	2.4	0.20
Attention retention system	24	7.2	0.59
Face recognition	2.25	0.675	0.05
Gesture recognition	2	0.6	0.05
Eye movement recognition	1.5	0.45	0.04
NFC communication	2	0.6	0.05
Wireless charger	10	3	0.24

**Table 4:** Carbon footprints at different levels of intelligence.

Level	Automation Function	Connection Function	Other Factors (e.g., Hardware Quality)	Carbon Footprint (CO_2_ g/km)	Cost (€)
Basic	80%	15%	5%	234	19.95
Intermediate	73%	20%	7%	468	39.89
Advanced	61%	33%	6%	558	47.56

**Table 5:** Estimation of carbon footprint reduction through 3D printing in EVs/AVs.

Factor	Material Efficiency	Lightweighting	Customization	Local Production	Recycling and Circular Economy	Energy Efficiency
Moderate	20%	10%	5%	15%	5%	5%
Aggressive	30%	15%	10%	20%	5%	10%
